# ‘Cooking is for everyone?’: Exploring the complexity of gendered dynamics in a cookstove intervention study in rural Malawi

**DOI:** 10.1080/16549716.2021.2006425

**Published:** 2021-12-10

**Authors:** Jane Ardrey, Kate Jehan, Nicola Desmond, Caroline Kumbuyo, Kevin Mortimer, Rachel Tolhurst

**Affiliations:** a Department of International Health LSTM, Liverpool School of Tropical Medicine, Liverpool, UK; b Department of Public Health, Policy and Systems, Institute of Population Health, University of Liverpool, Liverpool, UK; c Malawi-Liverpool-Wellcome Trust Clinical Research Programme, Blantyre, Malawi; d Department of Clinical Science LSTM, Liverpool School of Tropical Medicine, Liverpool, UK

**Keywords:** Malawi, cookstove, gender, photovoice, air pollution

## Abstract

**Background:**

Household air pollution (HAP) resulting from cooking on open fires has been linked to considerable ill-health in women and girls, including chronic respiratory diseases, and has been identified as a contributor to climate change. It has been suggested that cleaner burning cookstoves can mitigate these risks, and that time saved through speedier cooking can lead to the economic empowerment of women. Despite these and other potential advantages of cookstoves, sustained use is difficult to achieve.

**Objective:**

We used qualitative methods (focus groups, interviews, observation) and the participatory methodology Photovoice in order to inform a deeper understanding of gendered social relationships within the Cooking and Pneumonia Study (CAPS) in rural Malawi.

**Methods:**

Over five CAPS villages, forty women and ten men were recruited for Photovoice activities, including image collection, village-level focus group discussion and interviews. Data were also collected from interviews with village-based community representatives.

**Results:**

This study facilitated a rich exploration of context-specific gendered household roles and power relations which found that there was space for contestation in seemingly entrenched and ‘traditional’ household responsibilities. The results suggest that the introduction of cookstoves through CAPS provided a focus for this contestation. It was evident that men and children also cooked, and that cooking played a central role in the gendered socialisation of children. However, there were no indications that time saved resulted in the empowerment of women.

**Conclusion:**

Our findings suggest that dominant narratives of the links between gender and cookstoves are often reductive and fail to reflect the complexity of gender power relations. The use of qualitative methods incorporating Photovoice helped to facilitate an alternative ‘bottom-up’ view of cookstove use which demonstrated that while cookstoves may disrupt gendered relationships in target communities, positive impacts for women and girls cannot be assumed.

## Background

### The rationale for promoting cookstoves

In light of the United Nations Climate Change Conference 2021 (COP26) and other climate emergency initiatives, there is renewed interest in the potential of improved cookstoves to replace open fire cooking and therefore reduce household air pollution [1]. The links between climate change mitigation and open fire cooking include concerns about deforestation, global warming and direct impact on increased rates of glacier degradation [2]. Cookstoves are also promoted due to their potential to improve health, with suggestions that women are particularly at risk due to their gendered roles as primary household cooks [3]. This promotion occurs even though no health-based trial of cleaner cooking interventions, including CAPS [4], has shown that cookstoves alone improve health [5]. In addition, practitioners and advocacy organisations often promote the use of cookstoves as empowering, because they are expected to enable women and girls to participate in education and employment activities through reducing the time burden of reproductive work [6].

### The scope of the problem

Recent estimates show that four billion people worldwide lack access to healthy, clean cooking energy and while progress has been made in some countries, growing populations and technical and resource limitations, are hampering advancement in sub-Saharan Africa [7]. Sustainable Development Goal (SDG) 7 is ‘universal access to affordable, reliable, sustainable, and modern energy services’, including a specific focus on clean cooking solutions [8] and has been linked with SDG 3 good health and well-being, SDG5 gender equality and SDG13 climate action [9].

In Malawi, 95% of people cook using biomass fuels [10] and as is the case in other low-resource settings, many will depend on open fire cooking or inefficient cookstoves for some time to come as access to cleaner fuels such as electricity continues to be limited [6]. Malawi has high levels of poverty and inequality with a national poverty rate of 51.5% in 2016 and limited livelihood opportunities for many [10]; the national political economy continues to be shaped by ‘colonial power relationships’ [11].

### Gender and cookstoves

Unequal gender relations also compounded by (post)colonialism [12,13] further reduce economic opportunities and independence for women, the majority of whom rely largely on small-scale agriculture and petty trading [10,11] In Malawi the colonial legacy of removing ownership of ‘customary land’ from Malawians to European, underpins modern day agricultural economy to deleterious effect [14]. The postcolonial legacy in Malawi of a ‘crude dualistic’ understanding of gender has also reinforced ‘traditional’ gender roles to the disadvantage of women [11]. It has also been suggested that the challenges to achieving the ‘empowerment’ of women in the Global South are exacerbated by political and economic power imbalances, that have arisen from colonialism, and are maintained by contemporary social processes including globalisation [12].The links between cookstoves and economic development including the economic empowerment of women, have been problematised [6,15] and it is recognised that defining and measuring women’s empowerment is difficult [16,17]. However, the narrative that equates cookstoves with positive outcomes for women persists and remains a driver of cookstove promotion [18]. Such narratives tend to over-simplify the context-specific power relationships that shape the opportunities offered by technology. These power relations include those between women and men which are complex, impact on economic outcomes in various ways, and are revealed by the division of tasks inside and outside the home and by ascribing different gendered role ability and suitability [19].

Winther et al. also suggest that dominant narratives within gender-related energy discourses often fail to recognise and tackle the underlying socio-economic constructs that lead to inequitable power relations between women and men which undermines female empowerment objectives [20]. A useful distinction with regard to outcomes is to consider the condition and position of women and men, with the former referring to the current concrete circumstances in which they live, the latter to the position of women in society relative to that of men [21]. Whereas receiving a cookstove may in some circumstances improve the condition of women by lessening their workload, changing the position of women is only possible if the way that ‘gender determines power, status, and control over resources’ is considered [21].

### Summary

Barriers to achieving the potential benefits of cookstoves include inconsistent use, the tailing off of use over time, and concurrent use of cleaner cooking technology with open fire and inefficient cookstove use (stacking) [22]. Nevertheless, as the climate crisis drives the pace of clean cooking implementation, the rollout of cookstove initiatives that are promoted as a panacea both for household air pollution and gender inequalities, is continuing apace. This is despite an increasing body of evidence that they do not deliver the anticipated health benefits [5]. Therefore an exploration of the complex nature of the interaction between gender and cookstove use in the context of a cookstove trial, is warranted. To deepen this analysis our study was informed by two perspectives. First, that cooking forms an important and time-consuming part of what Moser characterised as women’s ‘triple burden’ of productive, reproductive and community management roles [23] and is usually considered to be part of the unequal gender division of labour. Second by Meah’s suggestion that the household cooking environment is a ‘contested space for men and women’ that can provide women with ‘opportunities to exercise agency and resistance’ [24].

## Methods

### Study setting

This paper analyses complex gendered dynamics with regard to cookstove use that were explored through the use of qualitative methods and the participatory methodology Photovoice, nested within the Malawi-based Cooking and Pneumonia Study (CAPS). We carried out this qualitative study under the aegis of the Malawi-Liverpool-Wellcome Trust Clinical Research Programme (MLW) at their field site in Chikwawa, rural Malawi. MLW has been based in Blantyre, Malawi since 2002, works closely with the Malawi Ministry of Health and College of Medicine, and has carried out a range of health-based research in urban, peri-urban and rural locations, including several studies in Chikwawa [25].

CAPS was a randomised controlled trial which compared the effects of open fire cooking and a cleaner burning biomass-fuelled cookstove, on incidence of pneumonia in children under 5 [4]. CAPS was conducted in 150 villages in two districts, Chilumba in the North and Chikwawa in the South of Malawi, between 2013 and 2016, with over 10,000 children enrolled [4]. Eligible families with children under-5, were randomised into control and intervention arms of equal size. Participants in intervention villages were provided with cleaner burning biomass cookstoves, and a solar panel to power an integral fan and those in control villages continued to cook on open fires for the 2-year duration of the study [4]. CAPS found no evidence that the introduction of cleaner cookstoves led to a reduction in the risk of childhood pneumonia [4]. The authors suggested that a more holistic approach to tackling clean air inside and outside the home, combined with highly acceptable clean cooking solutions, is required to provide health benefits [4].

### Study design

This paper reports on gender-related findings determined by exploring the following question: ‘How and why do families in CAPS villages use the intervention cookstove and how is this shaped by gendered insecure livelihoods and being part of a highly researched community?’ Health-related findings are reported elsewhere [26]. Observation, focus groups, interviews, and the participatory methodology Photovoice were combined in phased qualitative research carried out between April and November 2016. Due to limited resources and the close collaborative relationship between the Liverpool School of Tropical Medicine and MLW, it was decided that this qualitative sub-study of CAPS should be carried out at the MLW Chikwawa site only.

The first author (JA) is a white European female and led the qualitative research study as part of her PhD research. At that time, she was employed as a Programme Manager for the BREATHE-Africa Partnership, and in that role had visited both CAPS sites and was known to the CAPS field team. She co-ordinated all activities working closely with the CAPS Qualitative Research Assistant (CK) who is a female Malawian and was studying for a social science qualification at the University of Malawi. CK was an established CAPS fieldworker employed since the study inception.

Employing a broadly constructivist approach [27] we used qualitative methods and the participatory methodology Photovoice to focus closely on meanings and interpretations of gender linked with cooking within the CAPS context, in order to inform a deeper understanding of gendered social relationships within cookstove interventions.

A constructivist approach, with an emphasis on meaning in context, and reflexivity of the researcher, necessitates the use of qualitative methods that facilitate a deep understanding of a specific socio-cultural context and how participants construct their reality [28]. In our study, consideration of the positionality of JA as a potentially powerful outsider, also led to the exploration of critical theory and the use of feminist and participatory methodologies that may promote greater equality between researchers and research participants [29]. Photovoice is a participatory action research methodology which entails participants capturing and discussing images about their own community strengths and challenges, and was developed from Wang and Burris’s work with Chinese village women in the 1990s [30].

The methodology is rooted in Freire’s concept of critical consciousness [31], contemporary approaches to documentary photography, and feminist inquiry [32]. Wang suggests that Photovoice can be characterized as a feminist methodology as it incorporates the subjective experience of women, recognises the importance of their everyday experience, and focuses on improving their health and wellbeing [32].

### Sampling

The study took place in April, July and November 2016 and participants can be divided into two groups: CAPS trial participants and Community Liaison Team (CoLT) members. Although the emphasis was on the perceptions and meanings of CAPS participants, CoLT members were included due to their important intermediary role in CAPS.

#### CAPS participants

CAPS trial participants were from five intervention villages purposively selected from the Chikwawa study site. Villages were selected for maximum variation [33], criteria included village size, geographical location including access to roads and trading centres, and availability of health services and schools. In overview, activities with CAPS participants carried out in each of the five villages were: observation of a cooking session; Photovoice training, image collection, and focus group; three semi-structured interviews with Photovoice and observation participants.

For observation sessions, CK liaised with the CoLT member of each of the villages to recruit a volunteer female household cook.

For Photovoice, ten participants were recruited from each village, two men and eight women and took part in Photovoice training, image collection and focus group discussions (FGDs); one per village. Three observation and Photovoice participants from each village were also interviewed individually. The primary selection criterion for observation and Photovoice participants was gender, due to the role of women in this context as primary household cooks, and in recognition of unequal intrahousehold power dynamics that privilege men [34]. Other purposive selection criteria included female heads of households and age. In two villages, observation participants also participated in Photovoice activities as shown in Table 1. Table 2 shows the age range and stated occupations of Photovoice participants.

**Table 1.** CAPS village participant sampling

CAPS Village Participants and Activities										
	*Village*									
	*1*		*2*		*3*		*4*		*5*	
	*Gender*									
	*F*	*M*	*F*	*M*	*F*	*M*	*F*	*M*	*F*	*M*
CAPS participant										
Observation	1		1		1		1		1	
Photovoice image collection	8	2	8	2	8	2	8	2	8	2
Photovoice FGD	8	2	8	2	8	2	8	2	8	2
Photovoice interview	1	1	2		2		1	1	1	1
Observation interview					1		1		1	
Photovoice/observation interview	1		1							
CoLT member										
Interview		1	1		1			1		1

**Table 2.** Details of photovoice participants

	Gender	Age range	List of stated occupations
Village 1			
	Female	24–32	Shareholder at Kasinthula sugar co-operative (2 female and 1 male) Mini-bus conductor Sells rice Subsistence farmer (3 as primary occupation and 1 as secondary)
	Male	28–50	
Village 2			
	Female	20–65	Runs bicycle hire business Casual labourer Subsistence farmer (7 as primary occupation and 3 as secondary)
	Male	23–32	
Village 3			
	Female	23–38	Sells Doughnuts Sells maize Builder Guard Subsistence farmer (6 as primary occupation and 2 as secondary) Pastor
	Male	46–51	
Village 4			
	Female	18–35	All – primary occupation of subsistence farmer Owns a shop Charcoal burner
	Male	30–36	
Village 5			
	Female	20–49	Sells doughnuts Owns a shop Pump attendant (Kasinthula) Sells phones Commercial farmer Subsistence farmer (6 as primary occupation and 3 as secondary)
	Male	23–53	

#### CoLT members

The second participant group were CoLTs from each of the five villages. CoLT members are community members recruited by MLW with the aim of identifying and reporting the concerns of research participants [35]. They also assist with logistical arrangements for, and communication about, MLW research activities. The selection of CoLT members was not purposive in that each village had only one CoLT member. However, the group contained a gender mix, two women and three men, all were well established in their communities as older married people with children and had several years’ experience as CoLT members.

### Data Collection

#### CAPS participants

Observation sessions, one in each village, involved a female participant preparing lunch observed by JA and CK. As is common in villages in Chikwawa this activity took place outside. JA developed a framework of: family; food; fuel; finance to guide collection and analysis of fieldnotes. The aim of these observation sessions was for JA to gain further familiarity with the context for cooking activities to inform the design and analysis of Photovoice activities.

A pilot study of Photovoice methodology was conducted to assess the feasibility and efficacy of using the methodology in this context as described in a separate manuscript [36] and the findings informed the three stages of Photovoice in this study, that is: Photovoice training and image collection; focus group discussion; semi-structured interviews, carried out in each of the five villages. First, Photovoice participants were trained as a group on how to use the digital camera and how to collect images safely and ethically, by a local photographer. CK introduced the concept of Photovoice, the aims of the study and the practical arrangements: each participant was asked to collect 50 images over 5 days to illustrate: what they ate; how this food was cooked; who cooked the food; who they cooked and ate with. They were also advised that they could take 20 images of their family and friends for their own use. Short practice sessions were then held in each village.

Second, after 5 days, the cameras were collected and taken to the nearest town to be processed. The first 75 images on each camera were printed off and distributed to the photographers at village-level focus groups. Focus groups took place in local village facilities such as schools or communal buildings and the SHOWeD acronym developed by Wang and Burris was used to encourage discussion, that is: what do you see here; what’s really Happening here; how does this relate to our lives; why does this problem or this strength exist; what can we do about this [37]? The images selected by the participants were spread out on large sheets of paper on the floor and grouped into discussion themes. For example, cooking using an open fire. Third, in November 2016, semi-structured interviews were carried out with selected Photovoice participants and the observation participants, informed by interim analysis. Interviewees were asked: about food they had cooked the previous day including where it was obtained, how it was cooked and who shared it; to reflect on any health impacts of the intervention cookstoves; and how any saved time from faster cooking was used.

#### CoLT members

Each of the village CoLT members were interviewed and asked to share their understanding: of any benefits or negative impacts of CAPS from the point of view of the participants; family structures in Chikwawa and how family members spent their time; of how good health was defined by people living in Chikwawa.

### Data analysis

JA co-ordinated and was present for all qualitative study activities including Photovoice sessions. CK led on the selection of participants, facilitated Photovoice training and FGDs, interviewed CoLT members and CAPS participants, all in Chichewa.

All focus groups and interviews were recorded using digital recorders. Translation and transcription were completed by CK with assistance from the MLW Translation and Transcription Team. The data analysis approach was thematic and completed by JA using the framework approach where matrices are used to facilitate a visual comparison of data [38]. Our personal experiences can shape how we read and code data. When coding, JA worked reflexively, alert to any assumptions she had regarding accounts within the data. Consistent with a constructivist approach, attention was paid to how participants accounted for and made sense of their own experiences. Codes applied were data-driven [39] in the first instance, rather than conceptual, in order to stay close to the data, with more conceptual analysis occurring later on as JA interpreted the data.

The use of participant collected images showing family and neighbours in a context where many activities including cooking take place outdoors, presented ethical challenges throughout this study. Accordingly, the importance of gaining verbal approval for taking images was stressed during Photovoice training. The approach taken in this study, informed by the previous pilot study [36], was to employ ‘situated visual ethics’ [40]. Situated visual ethics are context relevant, flexible and critical means of ethical decision making that take into account the different perspectives and power differentials within the research process (40). We aimed to avoid ‘condescending ethics’ and the ‘othering’ of the Photovoice participants which may have limited their contribution in ‘knowledge production processes’ [40], through careful consideration of how individual images were used to represent and advocate for Chikwawa residents. This includes the four images used in the paper for which specific approval was obtained from photographers and subjects in August 2021.

## Results

Analysis and interpretation of the data showed that complex negotiations within gender relations are integral to cookstove use, and to how time saved through faster cooking is spent. These results are detailed here through two inter-linking themes. Firstly, negotiating cooking in the household division of labour, with sub-themes: maintenance and contestation of gendered norms and changing gender roles. Secondly, uses of saved cooking time are not transformative.

### Negotiating cooking in the household division of labour

#### Maintenance and contestation of gendered norms

Discussions with CoLT members and CAPS participants demonstrated the predominance of narratives of fixed gendered divisions of labour for women and men, and barriers to challenging the status quo.

For example, a male CoLT member described the demarcation between the household roles of men and women:

Men, their job is, if they are employed …, they go to work and then they come back, or they go to work in the farm. They are supposed to cut down trees, building kraals, that’s work, or digging toilet. Women are supposed to cook food, fetch water for bathing, wash clothes, mopping in the house and making sure that all the children have taken their bath. (CoLT member Village 1)

There are indications of resistance to deviations from embedded normative behaviour with risks for those who step outside their usual roles. As described by a female CoLT member:

Eeeeh the men don’t do the cooking they just stay … … Eeeeh it’s like they run away from this issue of cooking … they say that the cooking issue is about the woman and that if they do it, they will look foolish. They say that it is the ladies who love cooking. (Female CoLT member Village 3)

**Figure 1.**
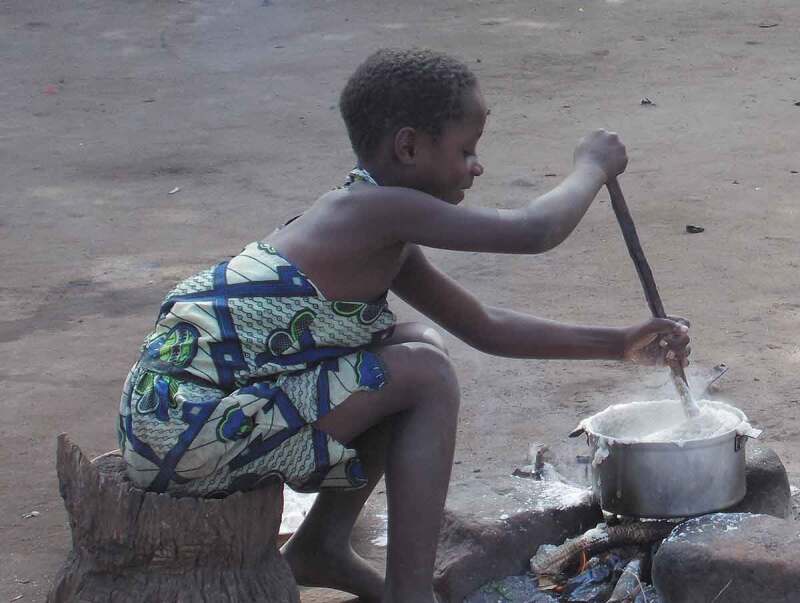
Learning the women's roles

Discussion of Photovoice images in FGDs and interviews provided a focus for exploration of how the status quo is maintained and to a certain extent contested. Referring to the image in Figure 1, an older male CAPS participant explained why he wanted to record this scene:

Yes, ee I was interested saying that aa the person who is cooking *nsima* here is a girl child and is learning some roles, so I took a picture. I thought it was interesting what she was doing; I knew that she was learning the women’s roles although cooking is for everyone, but this one has learnt well so I went closer to take the picture. (Male CAPS participant Village 4)

This image was collected by an older man who appears to be asserting the status quo by drawing attention to how the girl demonstrates ‘appropriate’ gendered conventional behaviour. However, his contradictory reference to ‘although cooking is for everyone’ suggests that he is aware of gender equality narratives regarding cooking.

In other cases, and mainly by women, the status quo is more clearly contested but not necessarily in ways that lead to a positive response from men. For example, a female CoLT member gave a more nuanced account of how ‘the world has turned around’ leading to women becoming involved in tasks designated as ‘male’ (such as cutting trees), but that asking a man to wash dishes would be seen as disrespectful. She concluded however, that while ‘women help men, men do not help women’. (CoLT member Village 2)

In a Photovoice focus group discussion a lively discussion illustrated how men and women incorporate and negotiate changing ideas of household roles and responsibilities. A female participant suggested that both men and women have burdensome responsibilities and therefore:

We should be working in a balanced manner, 50-50. If it is washing the plates, you finish the cleaning. If I am sweeping, you should mop, even if it’s cooking then you should portion. If it’s the piecework we should be going together. It should be 50-50. Is it clear? (Female CAPS participant Village 1)

Her proposed gender equitable approach was countered by a male participant who responded that some tasks are ‘shameful’ for men, making a clear link between gender identity and the unequal division of household roles, and suggesting that the status quo should be preserved.

#### Changing gender roles

**Figure 2.**
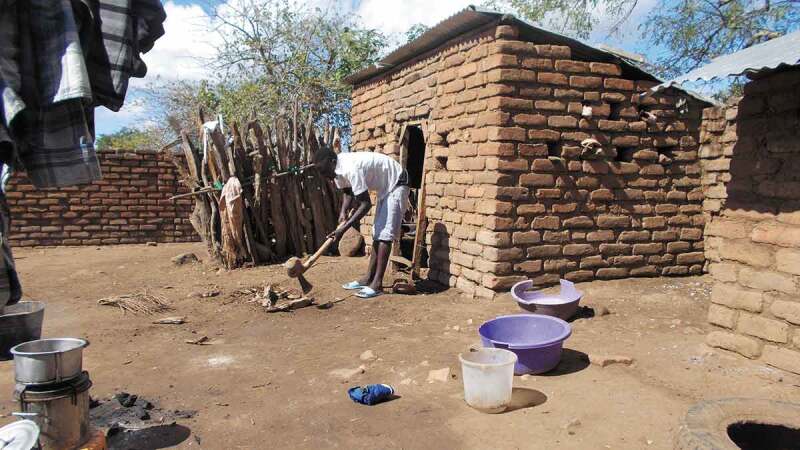
Boy chopping wood for cooking (after persuasion)

There is also contestation, ambiguity and indications of changing gender roles in descriptions of the involvement of children and young people in cooking activities described with reference to Photovoice images. The young woman who took the image in Figure 2 explained that she was trying to convince her brother that boys should assist with domestic work, and in this way, appears to be contesting gender ideology that demarcates household tasks as ‘women’s work’. As she described:

So, I took this picture to show that gender issues are difficult here because when we tell boys to help us with household chores, they refuse. They say that this doesn’t concern them because it is girls’ work. But this time … I told him to [do it] saying boys do this work most of the time. (Female CAPS participant Village 1)

It was clear from observation sessions and Photovoice activities, that it was common for children to cook in CAPS households, whether to assist preparing family meals, or for themselves using small amounts of found food, for example roasting corn kernels. However, it appeared that the responsibility for assisting with household cooking activities mostly lay with girls and boys refusing to help, was more acceptable to parents. A male CoLT member described how in some households, ‘boys are not allowed to work … girl children work like slaves, but other households try to get the boy to do work which is associated with girls’.

In their discussions of gendered household roles, CAPS participants and CoLT members make clear that they are familiar with the term gender and that it is associated with changes to gender roles introduced or promoted by outsiders. For example, a younger male CAPS participant (30-year-old shop owner) suggests that boys’ and girls’ household roles are interchangeable as now ‘with the coming of gender they should work equally’. The description of gender ‘coming’ indicates that this is a concept that comes from outside Chikwawa. CAPS participants also clearly refer to ‘gender’ as something that people do when acting against gendered norms. When explaining an image of her son helping her to collect water a Photovoice participant says that:

… he draws water for me and helps me because when they say gender, it means there is … the women’s jobs go to men, while the men’s jobs the women are also supposed to work on that. That is gender.^1^^1^It is noteworthy that this interviewee and other participants used the English term gender. In some cases, the Chichewa word *jenda* was used. This is defined as ‘gender equality/equal opportunities for women’ (Female CAPS participant Village 2)

**Figure 3.**
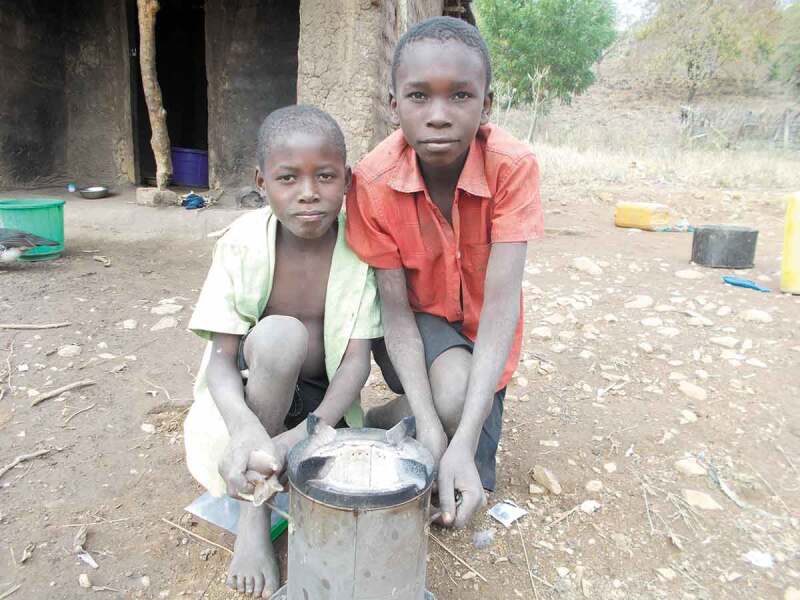
Boys posing with cookstove

Similarly, separate female participants use phrases such as: ‘he can cook, gender’ when describing an image of her son cooking and ‘we will do gender, they will be taking turns’ when describing sharing of household tasks between daughters and sons (Female CAPS participants Village 3). In this way, CAPS participants appear to be presenting a conceptualisation of gender as something new coming into the community and being directly related to a change in household roles. This is concurrent with Riley and Dodson’s suggestion that ideas of gender rights are perceived in Malawi as Western, modern and imposed from outside and that meanings of gender in the context are a ‘clear echo of colonial discourses’ [11]. The collection and discussion of several images of boys cooking also illustrate how boys were very interested in the intervention cookstoves and liked to use them, if allowed. For example, with reference to the image in Figure 3 a female CAPS participant says that:

He went to the bush for hunting [mice] and when he returned, he was saying he wanted to cook, “You should take a picture when I cook so that I should appear beside the cookstove”. And I told him that he couldn’t cook, then “alright just come and hold this side of the cookstove”. This child likes the cook stove very much. (Female CAPS participant Village 2)

Similarly, there are indications that CAPS stimulated interest in cookstoves and cooking amongst men. Throughout the trial there were reports that male interest in cookstove technology and particularly their potential as a power source for charging radios and phones had led to damage to cookstoves. The cookstove repair and replacement service was certainly used extensively, on average four times by each intervention household during the trial (4). There was also indication of ‘hidden’ use by men where they were able to cook inside without being observed due to the portability and low smoke emission of the cookstoves. A benefit of cookstoves explained by a male CoLT member was that men:

… are able, to cook when the stove is on the veranda … these men sometimes they are shy to cook in an open place so they can place it wherever they can be comfortable to use it. (CoLT member Village 3)

**Figure 4.**
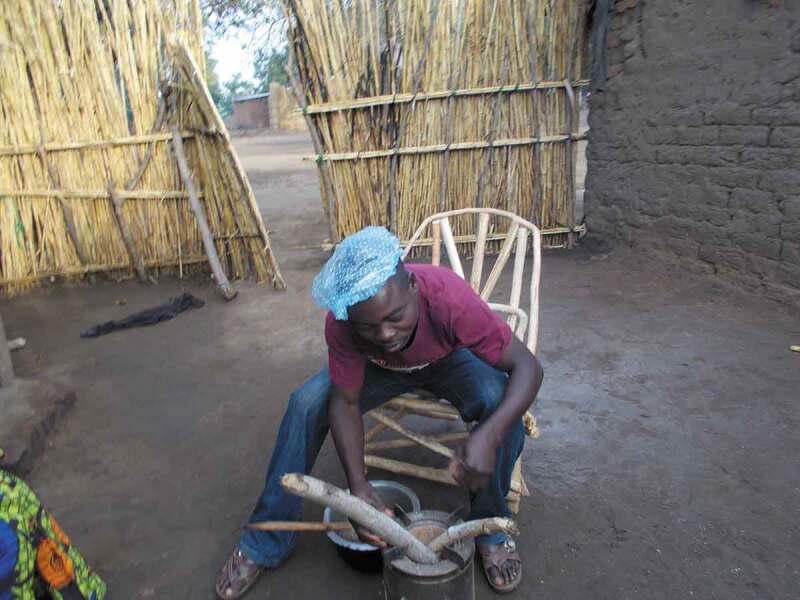
Man wearing wife's hat and pretending to cook

A male CAPS participant also suggests that the ease of use when cooking ‘enticed’ men to ‘help the ladies to cook’; they were attracted by the way the cookstove fan assisted with lighting and how the ‘volume’ could be easily turned up or down (Male CAPS participant Village 1). Some of the ambivalence and possible discomfort with men cooking is shown by a series of images collected by a male CAPS participant, in which he wears his wife’s hat and poses with the cookstove (see Figure 4). There appears to be an element of mockery in the image (researcher’s interpretation). This implicitly draws attention to gendered household cooking roles and the potential of engagement with new cookstoves to disrupt and shift existing norms.

### Uses of saved cooking time are not transformative

As described in the introduction, the idea that time-saved through faster cooking using cookstoves may have a transformative impact on women’s economic ‘empowerment’ is pervasive within the sector. When asked about the benefits of cookstoves, many CAPS participants clearly reported that they cooked quickly and therefore saved time, as in this description:

… the cook stoves are good … because they are fast, you are cooking fast, and you are like a hero (Female CAPS participant Village 3)

As demonstrated by the reference to being a ‘hero’, fast cooking appears to be appreciated by women because it allows them to fulfil their expected role as household providers of cooked food in a timely fashion. However, Photovoice discussions and interviews revealed that economic opportunities for both men and women were limited and that divisions in household roles were reflected in work outside the home. Women’s earning potential is also constricted by the necessity for women with children to stay close to home for childcare and household duties; the latter includes time-consuming food preparation and cooking duties. This is illustrated below:

For us to find money my husband is the one who runs here and there, maybe building houses, then maybe he will find *ganyu* [casual labour] to do maybe for 2 weeks or I week then he comes back. If it happens that I have found a [way to make] a little money then … it is just maybe to find salt [that is, for everyday expenses] (laughs). I maybe cook doughnuts, sometimes I do the same with small fish and sell (Female CoLT member Village 2)

Although it must be recognised that in gendered narratives of work, the role of women’s contribution is often overlooked, and women themselves may not always describe their productive activity as work [21], it is clear that obtaining paid employment in Chikwawa is challenging for both men and women. In rural Malawi, casual labour, referred to as *ganyu*, is an important but insecure form of low paid employment which developed in the post-colonial period as a form of labour exchange between poorer and more well-off households [13]. Bryceson suggests that *ganyu* is poorly renumerated and can deepen food insecurity for women and men, but that women are further disadvantaged as they are generally paid less, and their opportunities are limited by the expectation that they stay closer to home, due to ‘social propriety as well as childcare responsibilities’ [13].

Although these results show that female CAPS participants did participate in *ganyu*, particularly the poorest who ‘don’t even own goats ‘CAPS trial participant Village 2), women outlined different income earning opportunities by gender. Men were often involved in building tasks, whereas tasks open to women were seen as less skilled and therefore more poorly remunerated, for example, fetching water for moulding bricks (CAPS trial participant Village 3). A male CoLT member is clear that:

… women say that moulding bricks, building houses is for men, if you find a woman moulding brick then they say that the woman has no husband (CoLT member Village 5)

This suggests that women, sometimes do take up tasks such as moulding bricks and building houses, but generally these types of tasks are gendered, with more varied and potentially better paid roles reserved for men. Therefore, in this context, even if cookstoves did free up time for employment outside the home, paid work opportunities are constrained and unlikely to create economic empowerment for women.

However, when women were asked how they spent the time saved from faster cooking, they did not mention paid work outside the home, and seemed uncertain about why the question was being asked. Female cooks provided responses such as ‘I do other household chores’ like washing clothes or drawing water (CAPS trial participant Village 3); ‘I sleep on the mat – waiting for the evening to light the fire again and cook supper’ (CAPS trial participant Village 5). A male participant suggested that his wife might ‘go to fetch water, wash dishes, taking care just the way it happens at home’ (CAPS trial participant Village 4).

Time saved from fast cooking was therefore constructed as valuable, not because it freed up women for income generation, but because it allowed more time for household tasks, for rest or for leisure. In the context of days categorised by the ‘continual drudgery’ [41] of household work and care in a low-resource setting, this additional time was beneficial. However, while rest in such contexts may be helpful for women, it does not necessarily constitute empowerment. That is, it does not lead to a process of change in which women gain the ability to make strategic life choices [42]. In addition, the availability of work opportunities outside the home that might facilitate the economic empowerment of women as claimed by many cookstove implementors, was very limited.

Women’s emphasis on the use of saved time for ‘more of the same’ takes place in a challenging context of finding employment opportunities compatible with onerous household duties. This study showed that the uses of women’s freed time reflected these challenges and closely gendered social norms and inequities, and that the extra time acquired was used for rest and completion of household tasks, as opposed to seeking work outside the home. In summary, whilst cookstoves did have benefits for women’s ‘condition’ they did not have a transformative impact on their ‘position’ and instead highlighted pervasive and embedded gender inequalities inside and outside the home.

## Discussion

These results indicate that an emphasis on women as primary cooks combined with a narrative that equates cookstoves with female empowerment can obscure complex gendered household dynamics. These dynamics impact on cookstove use and the potential of cookstoves to reduce gender inequities. Data showed that household cooking is an everyday activity carried out mainly by women but also by men and children, although there are variations between households regarding the expected behaviour of boys and girls.

In their study of gendered household roles and food security in urban Malawi, Riley and Dodson found that the concept of gender was ‘constructed, reworked and resisted’ leading to a ‘distinct set of local associations with the word gender’ [11]. The authors (italicising the local understanding of gender) found differences between the views of men and women, in that the former took a paternalistic approach and associated practicing *gender* with helping women with their domestic duties. By contrast, women generally conceptualised *gender* as related to ‘positive social change’, where household responsibilities were more equitably shared. However, both men and women appeared to link ‘*gender*’ with being progressive and with modernity [11]. In our study, participants used both the English word gender or a Chichewa word *jenda*, which has a dictionary definition of ‘gender equality/equal opportunities for women’ [43]. Similar to Riley and Dodson’s findings however, it was clear that the local concept of gender was closely related to the maintenance and contestation of gendered norms within a specific context.

Relating these findings to the CAPS context, the clinical trial, the introduction of new cooking technology and interactions with MLW as an external, powerful organisation may have provided an opportunity for gendered norms to be challenged by the disruptive impact of the incursion of ‘modern’ cookstoves into the home. Further, this qualitative study and particularly the Photovoice activities appear to have encouraged the exploration of and expression of ambivalence about household cooking roles and ‘doing gender’.

That is not to say that CAPS and the introduction of cookstoves had a transformative impact on the ‘empowerment’ of women. The shifting of household roles is challenging; as West and Zimmerman delineate, the division of household roles is not inconsequential but is key to the maintenance of the ‘dominant and subordinate statuses’ of men and women [44]. Riley and Dodson also conclude that the promotion of gender equality in Malawi has been hampered by a failure to ‘connect global ideas with the grounded reality’ and an under-appreciation of context-specific meanings of gender [11] and this would also seem to apply to claims for female empowerment through cookstoves.

In the two common narratives expressed in relation to female empowerment and clean energy, women are either portrayed as victims trapped in drudgery and with little autonomy, as needing to be ‘empowered’. Or as transformative neoliberal actors who through their ‘empowered’ involvement in clean energy initiatives, facilitate access for themselves and others [20,45]. An alternative view is that there is a long-standing and influential cookstove development discourse which privileges technological, male, European ‘experts’ and downplays the expertise and needs of female cooks [46]. Our study suggests that social and material realities within cookstove interventions are complex and context-specific, and that a direct linkage between the introduction of Western technology and improved gender equality cannot be assumed.

In summary, the promotion of cookstoves as enablers of a simple linear pathway to financial independence of women is reductive, fails to reflect the complexity of gendered household roles and relationships, and the wider environment of precarity and scarcity which has its legacy in post-colonial inequity. The linkage of financial autonomy and ‘empowerment’ is also subject to question [47]. Such narratives pay insufficient attention to the inequal power relations between men and women that underpin discriminatory ‘socio-material structures’, are detrimental to women, and hamper equitable change [20]. As shown in our findings and suggested by Meah, women may be reluctant to surrender their household responsibilities and may defend their position by mocking men who step outside ‘breadwinning’ roles [24]. They may also take a strategic approach, referred to by Kandiyoti as a ‘patriarchal bargain’, by largely conforming to gendered norms to optimise security, while exercising ‘active or passive resistance’ [48].

Our results showed that women did not report or appear to seek, transformative economic activity outside the home with time saved from fast cooking. Concurrent with Avotri and Walters findings in Ghana, combining family care and economic responsibilities where opportunities for paid work are limited, is challenging in Chikwawa and may simply add to the already heavy workload of women with little benefit [49]. This leads to the conclusion that even if time saved through faster cooking was used to seek paid employment, it cannot be assumed that this would lead to ‘empowerment’. Such an outcome demands attention to both the ‘condition’ and ‘position’ of women [21]. While there are indications in our study that cookstoves were helpful for women and prompted discussion of the gendered household roles, the introduction of cookstoves did not appear to have a transformative impact on the power inequity between women and men.

## Conclusion

In summary, the results of this study suggest that an emphasis on women as the primary cook in cookstove interventions may not fully reflect the role of children and men in cooking activities. However, the responsibility for cooking largely remains a female one and power relations surrounding cookstove use are complex, contested and locally situated. Time saved through faster cooking was generally welcomed by women and there were also indications that they sought the greater involvement of men and male children in household activities, including cooking. The assumption that such changes would lead to ‘empowerment’ of women and ultimately greater gender equity, greatly understates the power that underpins gendered household roles and how both women and men might defend the status quo.

The use of qualitative and participatory methodologies that engage communities and explore the disruptive impact of cookstove interventions are key to approaches that enable the ‘co-production of knowledge and impact’ [50]. Photovoice facilitated a deeper understanding of locally situated cooking practices and perceptions, in line with calls for an emphasis on the ‘mundane’ and on an approach that incorporates the experiences and values of cookstove users [51]. The use of participatory methodologies to consult with women both about their priorities regarding their ‘condition’ (material needs) and their ‘position’ (power to negotiate these) [21], is necessary if cookstove implementors aspire to positive outcomes for women. Technological innovation is unlikely to promote empowerment for women without accompanying social and structural changes. In the settings where cookstove and similar technological interventions are implemented, this also depends on acknowledging and countering the pervasive legacy of colonial gendered inequality.

## Paper context

Burning biomass fuels in open fires for cooking has been linked to ill-health and the climate emergency. Claimed beneficial impacts of cleaner cookstoves on women’s health and empowerment are key factors in their promotion. In the context of a cookstove intervention, gendered and power-laden social norms related to cooking were found to be well-defined but with some flexibility. This qualitative study demonstrated that positive impacts for women and girls from cookstove interventions cannot be assumed.

## References

[1] Watts N, Amann M, Arnell N, et al. The 2020 report of The Lancet Countdown on health and climate change: responding to converging crises. Lancet. 2020. DOI:10.1016/s0140-6736(20)32290-x PMC7616803 33278353

[2] Jeuland MA, Pattanayak SK. Benefits and costs of improved cookstoves: assessing the implications of variability in health, forest and climate impacts. PLOS ONE. 2012;7:e30338. 22348005 10.1371/journal.pone.0030338 PMC3278415

[3] Simkovich SM, Goodman D, Roa C, et al. The health and social implications of household air pollution and respiratory diseases. NPJ Prim Care Respir Med. 2019;29:12. 31028270 10.1038/s41533-019-0126-x PMC6486605

[4] Mortimer K, Ndamala CB, Naunje AW, et al. A cleaner burning biomass-fuelled cookstove intervention to prevent pneumonia in children under 5 years old in rural Malawi (the Cooking and Pneumonia Study): a cluster randomised controlled trial. Lancet. 2017;389:167–13. PubMed PMID: 27939058. 27939058 10.1016/S0140-6736(16)32507-7 PMC5783287

[5] Mortimer K, Balmes JR. Cookstove trials and tribulations: what is needed to decrease the burden of household air pollution? Ann Am Thorac Soc. 2018;15:539–541. 29466681 10.1513/AnnalsATS.201710-831GH

[6] de Groot J, Mohlakoana N, Knox A, et al. Fuelling women’s empowerment? An exploration of the linkages between gender, entrepreneurship and access to energy in the informal food sector. Energy Res Soc Sci. 2017;28:86–97.

[7] Health Effects Institute. State of global air 2020. Health Effects Institute; 2020.

[8] IEA I, UNSD, World Bank, WHO. Tracking SDG 7: the energy progress report. 2020.

[9] World Health Organisation. Sustainable development goals for household energy. 2020 [cited 2020 Aug 12]. Available from: https://www.who.int/airpollution/household/sustainable-development-goals/en/

[10] The World Bank in Malawi Overview: World Bank. 2019. Accessed19 November 2019. Available from: http://www.worldbank.org/en/country/Malawi/overview

[11] Riley L, Dodson B. ‘Gender hates men’: untangling gender and development discourses in food security fieldwork in urban Malawi. Gend Place Cult. 2016;23:1047–1060.

[12] Medie PA, Kang AJ. Power, knowledge and the politics of gender in the Global South. Eur J Politics Gender. 2018;1:37–53.

[13] Bryceson DF. Ganyu casual labour, famine and HIV/AIDS in rural Malawi: causality and casualty. J Mod Afr Stud. 2006;44:173–202.

[14] Ng’ong’ola C. Malawi’s agricultural economy and the evolution of legislation on the production and marketing of peasant economic crops. J South Afr Stud. 1986;12:240–262.

[15] Rewald R. Energy and women and girls analyzing the needs uses and impacts of energy on women and girls in the developing world. 2017. Accessed 26 March 201. Available from: https://www.oxfamamerica.org/explore/research-publications/energywomen-girls/

[16] Raj A. Gender empowerment index: a choice of progress or perfection. Lancet Glob Health. 2017;5:e849–e50. 28755893 10.1016/S2214-109X(17)30300-5

[17] Harper CM, George R, D’Angelo R, et al. Gender, power and progress: how norms change. London: ALIGN/ODI; 2020. p. 2020.

[18] Clean Cooking Alliance: Livelihoods: Clean Cooking Alliance. 2019 [cited 2019 Oct 10]. Available from: https://www.cleancookingalliance.org/impact-areas/livelihoods/

[19] Agarwal B. “Bargaining” and gender relations: within and beyond the household. Feminist Econ. 1997;3:1–51. PubMed PMID: 7620908.

[20] Winther T, Ulsrud K, Matinga M, et al. In the light of what we cannot see: exploring the interconnections between gender and electricity access. Energy Res Soc Sci. 2020;60:101334.

[21] March C, Smyth IA, Mukhopadhyay M. A guide to gender-analysis frameworks. Great Britain: Oxfam; 2001. p. 144.

[22] Gordon SB, Bruce NG, Grigg J, et al. Respiratory risks from household air pollution in low and middle income countries. Lancet Respir Med. 2014;2:823–860. PubMed PMID: 25193349. 25193349 10.1016/S2213-2600(14)70168-7 PMC5068561

[23] Moser CON. Gender planning and development: theory, practice, and training. London: Routledge; 1993.

[24] Meah A. Reconceptualizing power and gendered subjectivities in domestic cooking spaces. Prog Hum Geogr. 2014;38:671–690.

[25] Malawi-Liverpool Wellcome Trust Clinical Research Centre. Chikwawa. 2019 [cited 2019 Jan 13]. Available from: https://www.mlw.mw/index.php/chikwawa.html

[26] Ardrey J, Jehan K, Kumbuyo C, et al. ‘Pneumonia has gone’: exploring perceptions of health in a cookstove intervention trial in rural Malawi. BMJ Glob Health. 2021;6:e004596. 10.1136/bmjgh-2020-004596 PMC8506864 34635550

[27] Lincoln YS, Guba EG. The constructivist credo. Walnut Creek (CA): Routledge; 2016.

[28] Cresswell JW. Research design: qualitative, quantitative, and mixed methods approaches. 4th ed. California: Sage; 2014.

[29] Ritchie J, Lewis J, and McNaughton Nicholls C, et al. Qualitative research practice: a guide for social science students and researchers. California: Sage Publications Inc. 2014.

[30] Wang C, Burris MA. Photovoice: concept, methodology, and use for participatory needs assessment. Health Educ Behav. 1997;24:369–387. PubMed PMID: 9158980. 9158980 10.1177/109019819702400309

[31] Freire P. Pedagogy of the oppressed. London: Penguin Books; 1996.

[32] Wang CC. Photovoice: a participatory action research strategy applied to women’s health. J Womens Health. 1999;8:185–192. PubMed PMID: 10100132. 10100132 10.1089/jwh.1999.8.185

[33] Patton MQ, and Patton MQ. Qualitative evaluation and research methods. 2nd ed. California: Sage Publications; 1990.

[34] Fingleton-Smith E. The lights are on but no (men) are home. The effect of traditional gender roles on perceptions of energy in Kenya. Energy Res Soc Sci. 2018;40:211–219.

[35] Nyirenda D, Sariola S, Gooding K, et al. ‘We are the eyes and ears of researchers and community’: understanding the role of community advisory groups in representing researchers and communities in Malawi. Dev World Bioeth. 2018;18:420–428. 28872746 10.1111/dewb.12163 PMC6491972

[36] Ardrey J, Desmond N, Tolhurst R, et al. The cooking and pneumonia study (CAPS) in Malawi: a nested pilot of photovoice participatory research methodology. PLoS One. 2016;11:e0156500. PubMed PMID: 27254291; PubMed Central PMCID: PMCPMC4890783. 27254291 10.1371/journal.pone.0156500 PMC4890783

[37] Wang C, Burris MA. Empowerment through photo novella: portraits of participation. Health Educ Q. 1994;21:171–186. 8021146 10.1177/109019819402100204

[38] Braun V, and Clarke V Using thematic analysis in psychology. In: Qualitative research in psychology. London: Routledge; 2006. p. 77–101.

[39] Clarke V, and Braun V. Successful qualitative research: a practical guide for beginners. California: SAGE; 2013.

[40] Ponic P, Jategaonkar N. Balancing safety and action: ethical protocols for photovoice research with women who have experienced violence. Arts Health. 2012;4:189–202.

[41] Chopra Daz E. No time to rest: women’s lived experiences of balancing paid work and unpaid care work. Brighton: Institute of Development Studies; 2017.

[42] Kabeer N. Resources, agency, achievements: reflections on the measurement of women’s empowerment. Dev Change. 1999;30:435–464.

[43] Oxford Dictionary. Chichewa-English: English-Chichewa. South Africa: Oxford University Press ORBIS (Pty) Limited; 2016.

[44] Zimmerman DH, West C. Doing gender. Gender Soc. 1987;1:125–151.

[45] Standal K, Winther T, and Danielsen K. Energy politics and gender. In: Hancock KJ, and Allison JE, editors. The Oxford handbook of energy politics. Oxford: Oxford University Press; 2018, 6 .

[46] Crewe E. The silent traditions of developing cooks. In: Grillo RD, and Stirrat RL, editors. Discourses of development: anthropological perspectives. Oxford; New York: Berg; 1997, 67 .

[47] Sen A. Gender and cooperative conflicts. Helsinki (Finland): UNU-WIDER; 1987.

[48] Kandiyoti D. Bargaining with patriarchy. Gender Soc. 1988;2:274–290.

[49] Avotri JY, Walters V. “You just look at our work and see if you have any freedom on earth”: Ghanaian women’s accounts of their work and their health. Soc Sci Med. 1999;48:1123–1133. PubMed PMID: 10220014. 10220014 10.1016/s0277-9536(98)00422-5

[50] Jenkins KEH, Stephens JC, Reames TG, et al. Towards impactful energy justice research: transforming the power of academic engagement. Energy Res Soc Sci. 2020;67:101510.

[51] Chatti D, Archer M, Lennon M, et al. Exploring the mundane: towards an ethnographic approach to bioenergy. Energy Res Soc Sci. 2017;30:28–34. PubMed PMID: S2214629617301962.

